# Draft Genome Sequence of the Chitin-Degrading Psychrotolerant Bacterium Pedobacter jejuensis TN23, Isolated from Antarctic Soil

**DOI:** 10.1128/MRA.00523-21

**Published:** 2021-07-01

**Authors:** Ahnna Cho, Yong-Joon Cho, Ok-Sun Kim

**Affiliations:** a Division of Polar Life Sciences, Korea Polar Research Institute, Incheon, Republic of Korea; b Department of Biological Sciences, Seoul National University, Seoul, Republic of Korea; c Research Institute of Basic Sciences, Seoul National University, Seoul, Republic of Korea; Montana State University

## Abstract

Pedobacter jejuensis TN23 was isolated from soil from Terra Nova Bay, Victoria Land, Antarctica. The assembled draft genome size is 4,795,808 bp, and it contains a total of 4,095 genes with 3,970 coding sequences, including genes putatively involved in the degradation of chitin.

## ANNOUNCEMENT

Pedobacter jejuensis TN23, a Gram-negative strict aerobe (1), was isolated from soil from Terra Nova Bay, Victoria Land, Antarctica (74°37′26.0″S, 164°13′49.2″E). A soil sample was collected on 3 February 2011, suspended (0.5 g/2 ml) in phosphate-buffered saline (PBS), and spread onto a Reasoner’s 2A (R2A) agar plate. The plate was incubated at 10°C for 2 weeks in an aerobic state, and then a single colony was transferred to a new R2A agar plate for pure culture. This strain showed 98.01% similarity with the type strain Pedobacter jejuensis THG-DR3 (2) by 16S rRNA gene typing using universal 27F-1492R primers and the EzTaxon-e database (3).

The genomic DNA of *P. jejuensis* TN23 was extracted from a culture in R2A liquid medium using a PowerSoil DNA isolation kit (Qiagen, USA). The concentration and purity were determined using a Qubit 2.0 fluorometer (Invitrogen, USA), and 1 μg of genomic DNA was processed to the next step. A Nextera DNA Flex library prep kit (catalog number 20018704; Illumina, USA) was used to prepare the DNA library for sequencing following the manufacturer’s protocol. The sequencing was performed using the Illumina MiSeq platform and generated 1,567,670 reads (2 × 250-bp paired-end format).

The raw data were cleaned using Trimmomatic v0.36 (4) with default parameters for adapter removal and quality trimming. The trimmed reads were assembled using SPAdes v3.1.2 (5) in standard mode with read error and mismatch correction. The final assembly yielded 45 contigs with a length of 4,795,808 bp, an *N*_50_ value of 316,667 bp, a GC content of 35.8%, and a genome coverage of 196.13×. Gene prediction and annotation were carried out using the NCBI Prokaryotic Genome Annotation Pipeline (6). Finally, a total of 4,095 genes were identified, including 3,970 coding genes, 3 rRNAs, 49 tRNAs, 3 noncoding RNAs (ncRNAs), and 70 pseudogenes.

The closest strain in the public genome databases was identified as *Pedobacter* sp. strain RP-3-11 by average nucleotide identity comparison (7), with a value of 80.09%. Several species of *Pedobacter* have been reported to survive in the Arctic and Antarctic environments (8, 9). They are known to have the ability to degrade chitin and cellulose in the soil (8, 10). The genome of *P. jejuensis* TN23 also has one chitinase (D7004_05180) and one chitin disaccharide deacetylase (D7004_02630) related to chitin degradation. While the optimal temperature of most chitin deacetylases in fungi is 50 to 60°C (11), the enzyme in this genome may have high activity at low temperatures as a consequence of host adaptation to low temperatures. Moreover, there are four cold shock proteins (D7004_00650, D7004_02470, D7004_11185, and D7004_17470) and six osmoprotectant-transporting proteins (D7004_11055 to D7004_11060); glycine betaine accumulation against low temperatures functions as an osmoprotectant. This report is the first genome sequencing result for a *P. jejuensis* strain, and the information about the enzymes that degrade chitin should provide insights into a soil ecosystem under extreme environmental conditions.

### Data availability.

The raw Illumina sequence reads were deposited in the Sequence Read Archive (SRA) under the accession number SRP260158. The genome sequences and annotations were deposited under the GenBank accession number RBEE01000000.1 and the BioProject accession number PRJNA494037.
